# Sublethal heat treatment promotes breast cancer metastasis and its molecular mechanism revealed by quantitative proteomic analysis

**DOI:** 10.18632/aging.203884

**Published:** 2022-02-12

**Authors:** Shujun Xia, Xiaoyu Li, Shangyan Xu, Xiaofeng Ni, Weiwei Zhan, Wei Zhou

**Affiliations:** 1 Department of Ultrasound, Ruijin Hospital, Shanghai Jiao Tong University School of Medicine, Shanghai, China; 2 Department of Ultrasound, Ruijin Hospital Luwan Branch, School of Medicine, Shanghai Jiao Tong University, Shanghai, China

**Keywords:** sublethal heat treatment, breast cancer, molecular mechanism, proteomics

## Abstract

Radiofrequency ablation (RFA) is a frequently used thermal ablation technique for breast tumors. The study aimed to identify the effect of sublethal heat treatment on biological function of breast cancer cells and reveal its potential molecular mechanism. The expression profile of dysregulated proteins in sublethal heat treated breast cancer cells was analyzed by quantitative proteomic analysis. The differentially expressed proteins in the sublethal heat treated breast cancer were identified. The potential biological functions of these proteins were evaluated. The proliferation and invasion ability of breast cancer cells were enhanced after sublethal heat treatment. The expression profile of proteins in sublethal heat treated breast cancer cells was abundant, and most of which were newly discovered. A total of 206 differentially expressed proteins were identified. Among them, 101 proteins were downregulated while 105 proteins were upregulated. GO and KEGG analysis indicated that various systems were involved in the process of sublethal heat treatment including cancer, immune system, et al. Immunohistochemistry staining showed that the expression of Heat shock protein 1B, NOB1 and CRIP1 was highly expressed while the expression of BCLAF1 was lower in sublethal heat treated group. The proliferation and invasion ability of breast cancer cells were enhanced after sublethal heat treatment. Sublethal heat treatment caused gene alterations in cancer and immune system. Heat shock protein 1B, NOB1 and CRIP1 were upregulated while BCLAF1 was downregulated in breast cancer after sublethal heat treatment.

## INTRODUCTION

Thermal ablation techniques have been accepted as alternative curative therapeutics to surgery for a wide range of tumors due to their advantages including shorter hospital stay and minimal invasiveness [1]. The aim of thermal ablation is to cause irreversible tumor cell damage by aggregating heat in the tumor, inducing cell apoptosis and coagulative necrosis. However, sublethal heat treatment may ablate a relatively small area, and lead to tumor residual, which causes recurrence and metastasis [2, 3]. In hepatic cell carcinoma, the recurrence rate after radiofrequency ablation (RFA) is higher than that after surgery [4]. Thus, in the present, thermal ablation techniques are mostly applied in malignancies with small tumor size, widely metastasized malignant tumors or in benign tumors for volume reduction [5–7].

RFA and high intensity focused ultrasound (HIFU) are frequently used thermal ablation techniques for breast tumors. RFA was regarded as a safe and promising minimally invasive treatment for breast cancer ≤2cm in diameter [6]. For benign breast tumors such as fibroadenoma, US-guided HIFU treatment has been confirmed as an effective noninvasive method and is well tolerated by the patients [8]. During the ablation process, lethal heat stimulation causes tumor cells collapsing, cell membrane breaking down, nucleus shrinking and organelles dissolution. In the previous study, the sublethal heat treatment would promote the metastasis of residual hepatocellular carcinoma cells via upregulating flotillin proteins [9]. However, the effect of sublethal heat treatment on breast cancer cells and its mechanism has not been clearly identified.

Mass spectrometric method based quantitative proteomics discovers and screens out all the deregulated proteins caused by certain factor. The high-throughput quantification of proteins combined with bioinformatic analysis would indicate cellular biological functions. In this study, we would explore the differential expression profile of proteins in sublethal heat treated breast cancer cells by using quantitative proteomics. The differentially expressed proteins in sublethal heat treated breast cancer cells would be identified and the potential biological functions of these proteins would be explored, aiming to reveal the potential molecular mechanisms that may involve in the sublethal thermal ablation.

## RESULTS

### Sublethal heat treatment promoted proliferation and invasion of breast cancer cells

**Figure 1:**
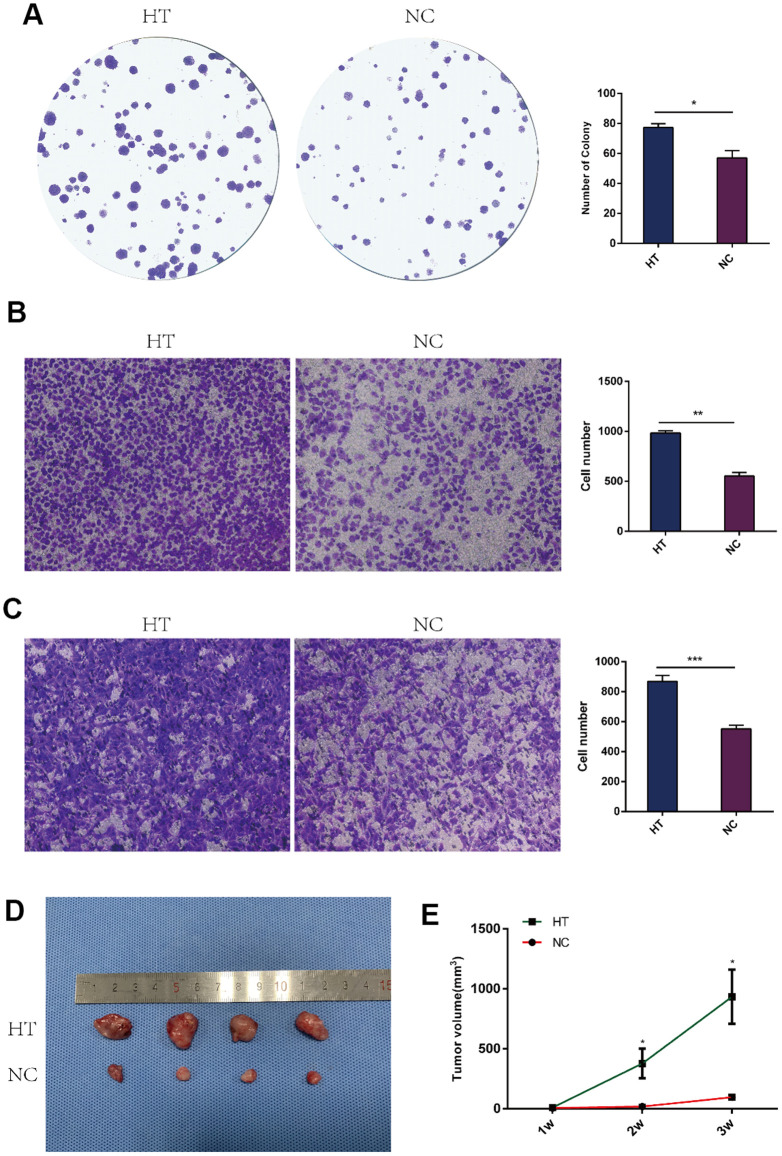
**Sublethal heat treatment promoted the proliferation and invasion of 4T1 cells.** (**A**) Colony formation assays were performed to assess the proliferation of 4T1 cells after sublethal heat treatment. The colonies were identified and counted. The number of colonies were presented as histograms. (**B**) Transwell assays were performed to determine the invasive ability of 4T1 cells after sublethal heat treatment. Representative images of invasive cells in the lower chamber stained with crystal violet. (**C**) Transwell assays were performed to determine the invasive ability of HUVECs after co-cultured with supernatant of sublethal heat treated 4T1 cells. The quantification of cell invasion was presented as invaded cell numbers. (**D**)The tumor grafts were showed (n=4 in each group) at the end of the 3rd week; (**E**) Tumor volumes were recorded and compared every week. All data were expressed as mean±SD of three independent experiments. HT=high temperature (45° C), NC=negative control (37° C). * indicates P<0.05, ** indicates P<0.01, *** indicates P<0.001.

*In vitro* and *in vivo* experiments were performed to study the proliferation and invasion abilities of 4T1 cells. The colony formation assay was done to evaluate the impact of sublethal heat treatment on 4T1 cells proliferation, which showed that there were more clones formed after sublethal heat treatment (Figure 1A). In addition, transwell invasion assay were performed on 4T1 cells and HUVECs to determine the invasive capacity. The number of 4T1 cells invading through the chamber in the sublethal heat treatment group was significantly more than that in the negative control group (Figure 1B). HUVECs were firstly co-cultured with the supernatant of 4T1 cells that underwent sublethal heat treatment, then the transwell invasion assay was performed. The treated HUVECs got more number of invasive cells than the control (Figure 1C). The subcutaneous tumor graft was constructed to determine the ability of tumor proliferation. Each group included four nude mice and the subcutaneous tumor grafts were compared (Figure 1D). The volume of the tumors was recorded every week. The tumor volume in the sublethal heat treatment group was larger at the end of the 1st, 2nd and 3rd week with comparison to the control (Figure 1E). These results indicate that sublethal heat treatment promotes proliferation and invasion in breast cancer cells.

### Detection of the expression of protein using LC-MS / MS

**Figure 2:**
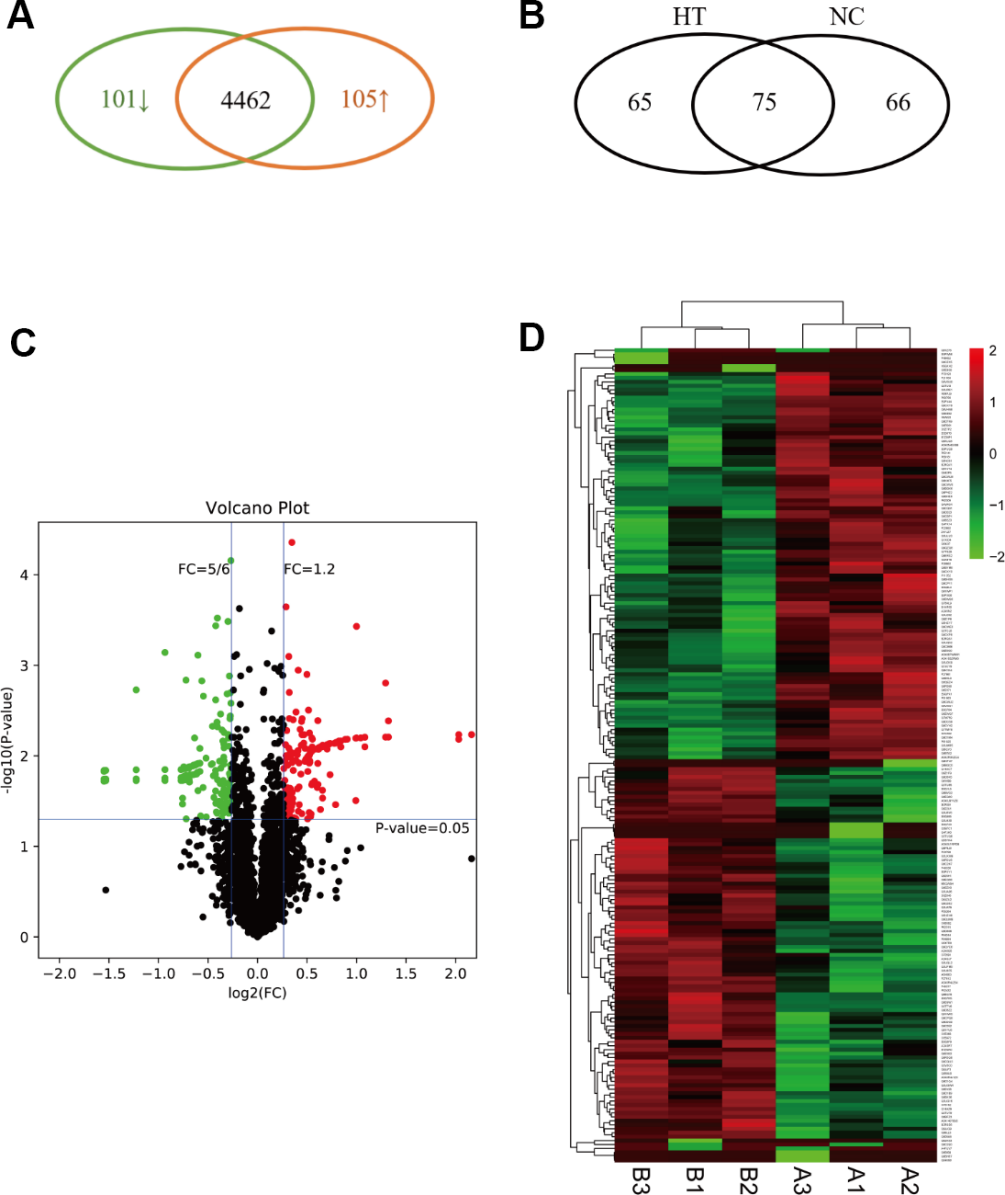
**Expression profile, definition and clustering of differentially expressed proteins in sublethal heat treated breast cancer cells.** (**A**) The Venn diagram indicated the upregulated and downregulated proteins in HT group vs. control group. (**B**) The Venn diagram indicated the within or without in HT group or control group. (**C**) Volcano plot defined up-regulated/down-regulated proteins. (**D**) Heatmap of DEP clustering analysis. **A** indicated control group and **B** indicated HT group.

To identify the differences in the proteomes of the sublethal heat treated breast cancer cells and the control group, we treated 4T1 cells in 45° C for 10 minutes and in 37° C as control (Supplementary Figure 1). Each group with three replicates were processed and analyzed using LC-MS/MS. A total of 206 DEPs were identified between the two groups, among which 101 proteins were downregulated while 105 proteins were upregulated (P<0.05) (Figure 2A). There were 65 proteins identified only in HT group, 66 only in NC group and 75 in both groups (Figure 2B). In the volcano plot, fold change less than 5/6 was defined as “downregulated”, and fold change more than 1.2 was defined as “upregulated” (Figure 2C). In the hierarchical cluster analysis, only the DEPs were taken into account(heatmap) (Figure 2D). The top 10 upregulated DEPs included: Heat shock protein 1B, Ribosomal protein L39, Tetratricopeptide repeat domain 13, RNA-binding protein NOB1, Beta-actin-like protein 1, etc.; the top 10 downregulated DEPs were: E3 ubiquitin-protein ligase HUWE1, Bcl-2-associated transcription factor 1, Cyclin-dependent kinases regulatory subunit 1, Histone H2A, etc. (See details in Supplementary Table 1).

### GO analysis of DEPs

**Figure 3:**
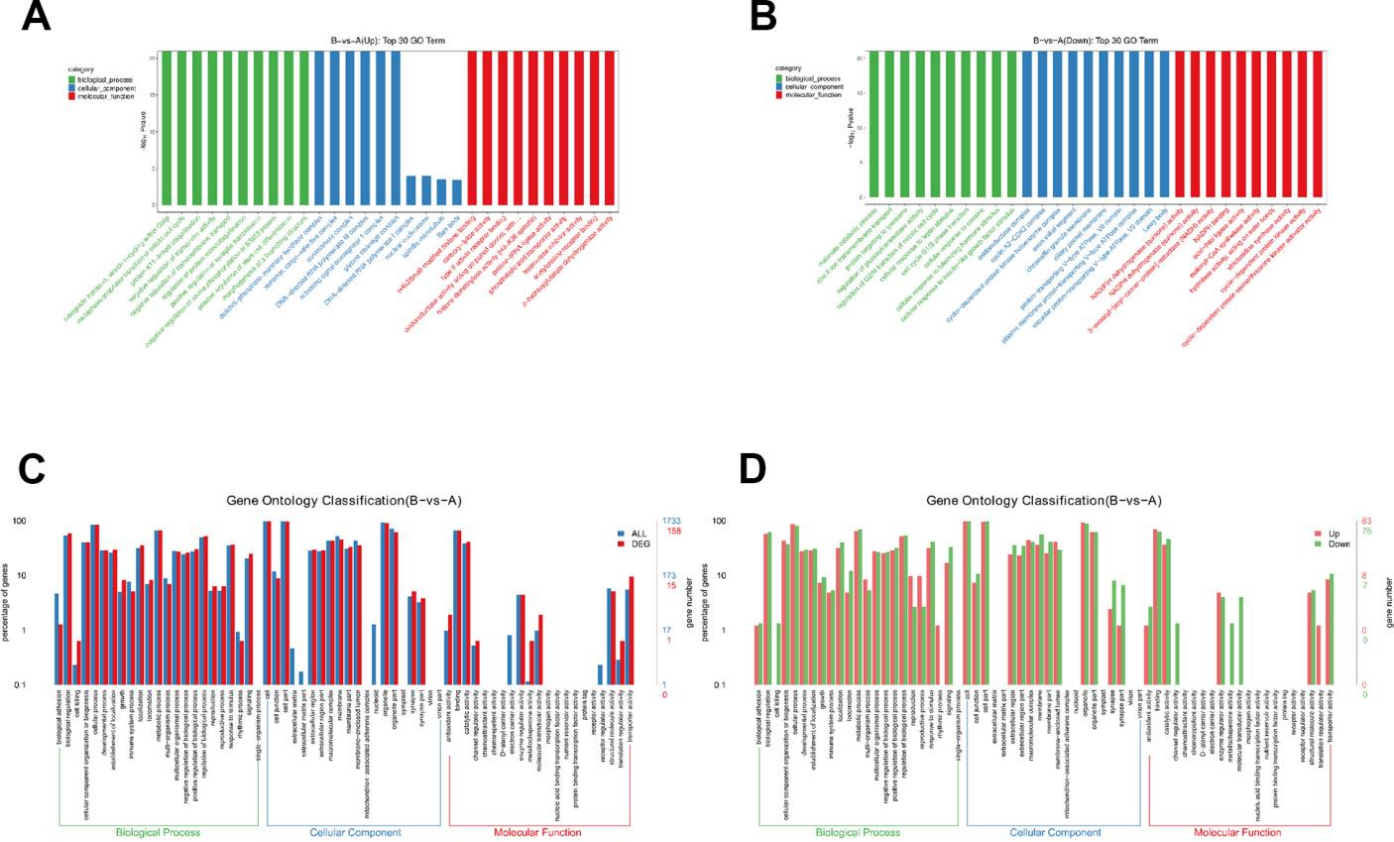
**Gene ontology analysis of DEPs.** (**A**) Go enrichment analysis results (upregulated). (**B**) Go enrichment analysis results (downregulated). (**C**) Comparison of the distribution of differentially expressed genes and all genes at Go Level 2. (**D**) Comparison of up-regulated and down regulated differentially expressed genes at Go Level 2.

Then, we performed enrichment analysis for GO based on DEPs. Go enrichment analysis top30 upregulated and downregulate were showed (Figure 3A, 3B), and detailed comparison group enrichment results showed in Supplementary Tables 2, 3. The distribution of differential genes and all genes at GO Level 2 were showed (Figure 3C), and detailed comparison group enrichment results showed in Supplementary Table 4. The distribution of upregulated and downregulated differentially expressed genes at GO Level 2 was showed (Figure 3D), and detailed comparison group enrichment results was showed in Supplementary Table 5. It was showed that biological processes including immune system and cellular process were involved during sublethal heat treatment. Cell part and organelle were mostly influenced cellular component. Binding and catalytic activity related genes were altered significantly.

### KEGG analysis of DEPs

**Figure 4:**
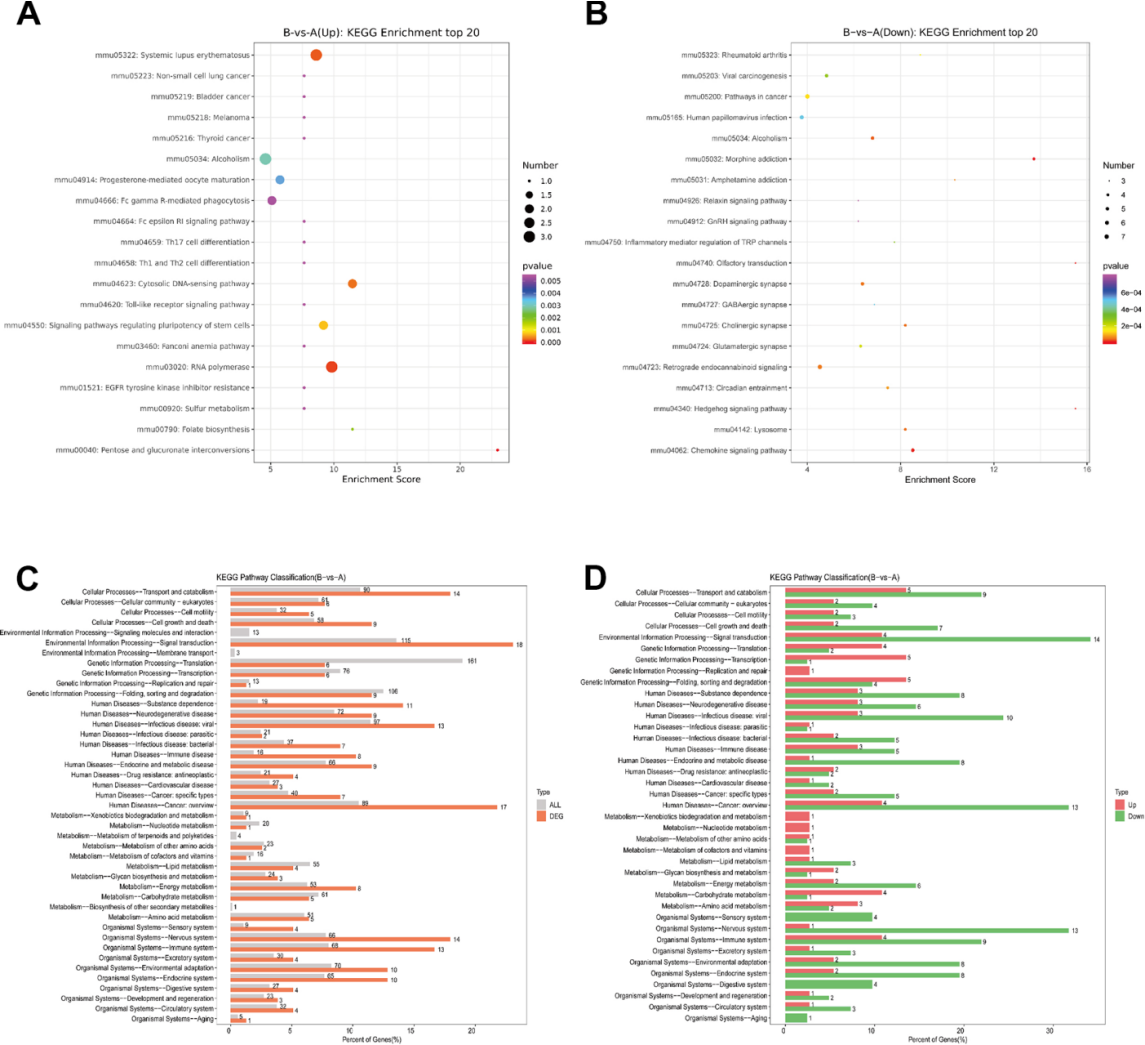
**KEGG pathway analysis of DEPs.** (**A**) Bubble Diagram of KEGG enrichment top20 upregulate. (**B**) Bubble Diagram of KEGG enrichment top20 downregulate. (**C**) Differentially expressed proteins / genes and all proteins / genes KEGG level2 horizontal distribution comparison chart. (**D**) Upregulate proteins / genes and downregulate proteins / genes KEGG level2 horizontal distribution comparison chart.

Then, we performed KEGG analysis based on DEPs. KEGG analysis top20 upregulated and downregulate were showed (Figure 4A, 4B), and detailed comparison group enrichment results showed in Supplementary Tables 6, 7. The distribution of differential genes and all genes at KEGG Level 2 were showed (Figure 4C), and detailed comparison group enrichment results showed in Supplementary Table 8. The distribution of upregulated and downregulated differentially expressed genes at KEGG Level 2 was showed (Figure 3D), and detailed comparison group enrichment results showed in Supplementary Table 9. It was indicated that DEPs were found in various systems including cancer, signal transduction and nervous system. And most of these DEPs were downregulated genes.

### PPI network of DEPs

### Protein-to-protein interaction (PPI) network construction and hub-molecule selection

**Figure 5:**
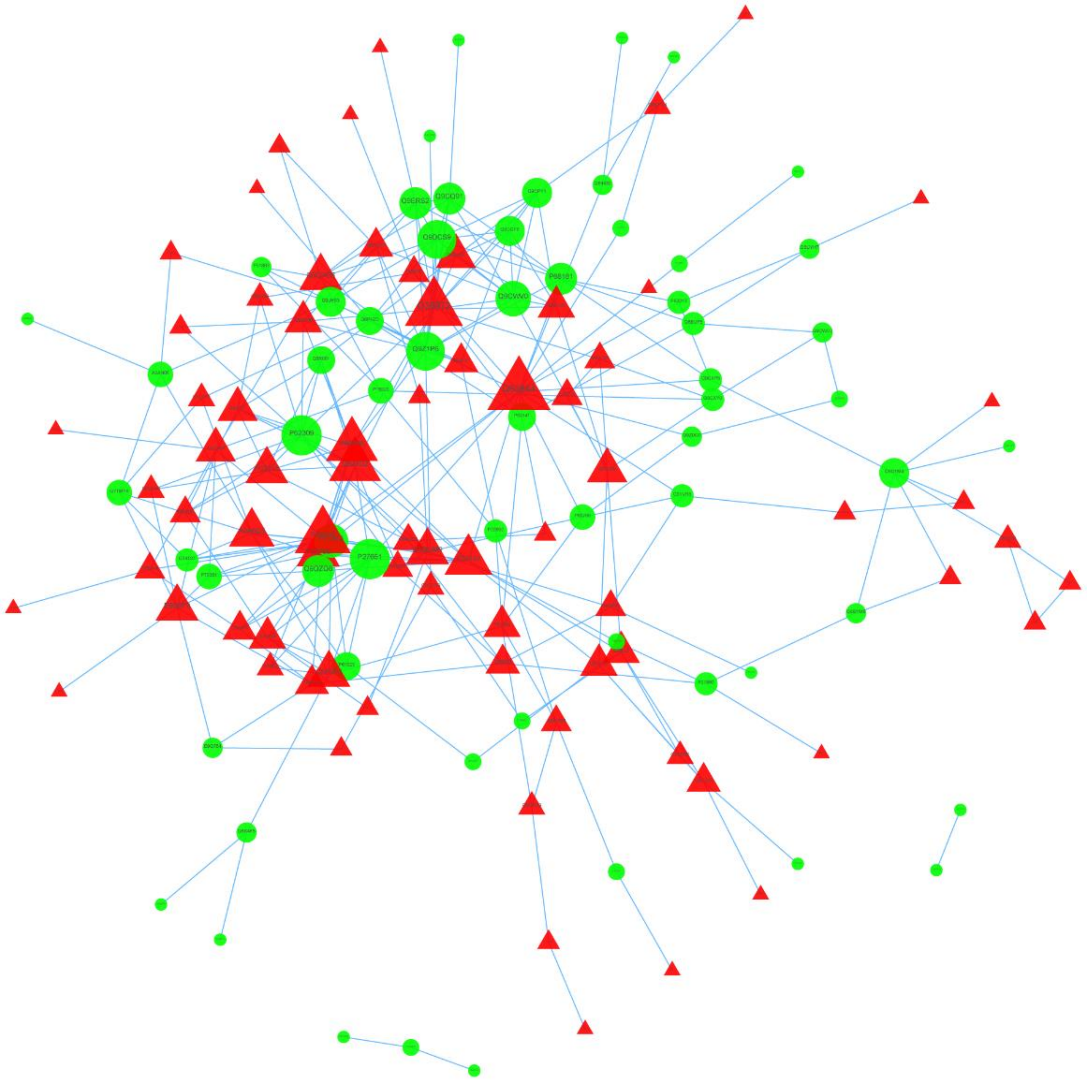
PPI network of DEPs.

We then used STRING to analyze protein-to-protein interaction (Figure 5). We identified nine core proteins that were closely connected to each other and changed drastically between and control patients. were all higher in HT group.

### Validation of DEPs *in vivo*

**Figure 6:**
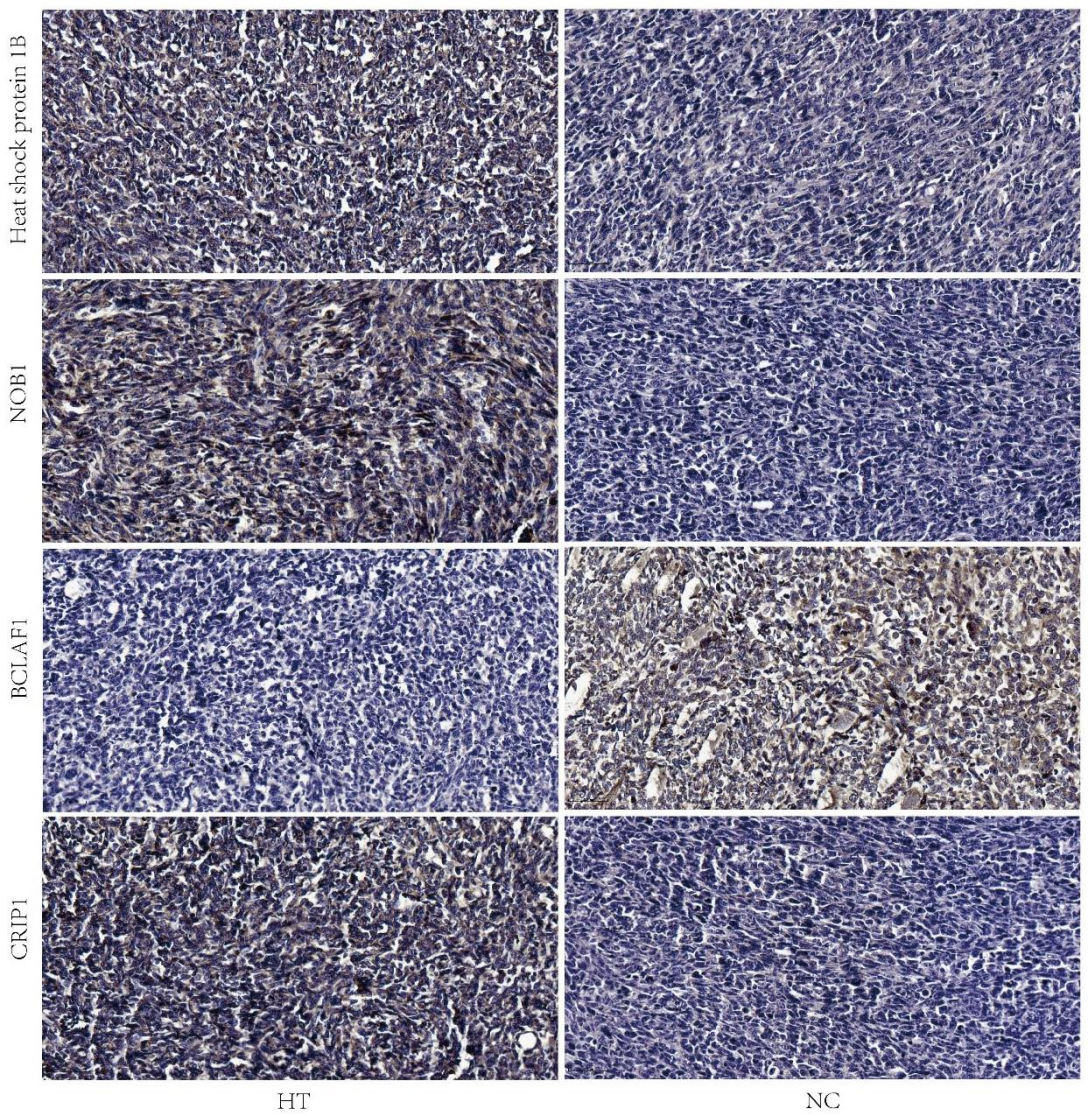
**Validation of the expressions of heat shock protein 1B, NOB1, BCLAF1 and CRIP1 by IHC.** Representative images of IHC analysis of the four proteins in HT and NC group were shown. The scale bar is 50 μm. HT=high temperature (45° C), NC=negative control (37° C).

Immunohistochemistry staining showed that the expression of Heat shock protein 1B, NOB1 and CRIP1 was highly expressed in HT group, however the expression of BCLAF1 was lower in HT group compared to NC group (Figure 6).

## DISCUSSION

Heat shock protein (HSP) is a kind of special protein produced by biological cells when stimulated [10]. It has biological activity and immune synergistic function [11]. It can protect the body or cells from or less damage, and plays an important role in maintaining the stability of the body itself. HSP1B is an important member of the small heat shock protein subfamily (sHSP subfamily) in the heat shock protein family [12]. This intronless gene encodes a 70kDa heat shock protein which is a member of the heat shock protein 70 family. In conjunction with other heat shock proteins, this protein stabilizes existing proteins against aggregation and mediates the folding of newly translated proteins in the cytosol and in organelles. It is also involved in the ubiquitin-proteasome pathway through interaction with the AU-rich element RNA-binding protein 1. The gene is located in the major histocompatibility complex class III region, in a cluster with two closely related genes which encode similar proteins. At present, many studies have confirmed that HSPB1 is closely related to tumor. HBV regulates the growth of hepatoma cells via mir-304-5p/ATF7/ HSPB1 signal axis [13]. HSPB1 rs2070804 polymorphism is associated with the depth of the primary tumor [14]. In our study we found that HT could promote the expression of HSPB1. We found that sublethal heat treatment can increase the proliferation and invasion of 4T1 cells. Therefore, we speculate that sublethal heat treatment can increase the proliferation and invasion of 4T1 cells by promoting the expression of HSPB1. Therefore, we will construct HSPb1 knockout and overexpression 4T1 cells to detect whether the regulatory effect of sublethal heat treatment on 4T1 is HSPB1 dependent.

NOB1 plays an important role in the biosynthesis of ribosome small subunit and 26S proteasome. NOB1 may be an oncogene, which promotes the proliferation of cervical cancer, papillary thyroid cancer and other malignant tumors [15–17]. miR-363 regulate the cell proliferation, migration and EMT through target the expression of NOB1 [18]. miR-612 suppressed the proliferation of cervical cancer by inhibiting the expression of NOB1 [15]. We found that the expression of NOB1 was higher than that of in control group *in vivo and in vitro*. NOB1 plays an important role in tumor growth and metastasis, so it may be a new target to prevent metastasis caused by sublethal heat treatment.

Guo H et al. had reported that Hspa1b is closely related to the occurrence of lung cancer [19]. NOB1 was a potential biomarker or target in cancer [20]. CRIP1 participates in regulation of proliferation, migration and invasion of breast cancer cells [21]. Bclaf1 participates in the regulation of breast cancer [22]. BCLAF1 is a protein rich in arginine serine RS domain which was located in the region of chromosome 6q22-23. In recent years, due to the increasing research on BCLAF1, it has been reported that BCLAF1 on cell surface plays an important role in ontogeny, cancer and other diseases by regulating the transcription and post transcriptional processing of specific genes and participating in the process of apoptosis, DNA damage response and differentiation [23–26]. However, the HT suppressed the expression of BCLAF1 indicated that HT may be partially inhibit the growth and proliferation of cells of 4T1 cells. CRIP1 is a member of the CRIP protein subfamily, which is considered as a new biomarker of osteosarcoma, prostate cancer and breast cancer.

In KEGG pathway analysis, we found that RNA polymerase signaling pathway was significantly increased in HT group. The increased transcription of ribosomal RNA gene (rDNA) catalyzed by RNA polymerase is a common feature of human cancer, but it is still unclear whether it is necessary to induce malignant phenotype, it has been reported that inhibition of RNA polymerase can activate p53 in the treatment of tumor, small molecule drug cx-5461 (cx-5461 is an effective small molecule rRNA synthesis inhibitor) can target rDNA transcription, thus selectively killing B lymphoma cells *in vivo*, while maintaining the survival of wild-type B cell population. The therapeutic effect is the result of p53 dependent apoptosis signal activation and nucleolus destruction. Human leukemia and lymphoma cell lines also showed a high sensitivity to this inhibition of rDNA transcription, which was dependent on p53 gene mutation [27]. Therefore, inhibition of RNA polymerase signaling pathway may inhibit HT induced tumor metastasis.

## CONCLUSIONS

Sublethal heat treatment promoted proliferation and invasion of breast cancer cells and caused gene alterations in cancer and immune system. Heat shock protein 1B, NOB1 and CRIP1 were upregulated while BCLAF1 was downregulated in breast cancer after sublethal heat treatment.

## MATERIALS AND METHODS

### Cell culture and heat treatment *in vitro*

Breast cancer cell line 4T1 was purchased from the cell bank of the Chinese Academy of Sciences (Shanghai, China). The 4T1 cells and human umbilical vein endothelial cells (HUVECs) were cultured in DMEM medium supplemented with HEPES (Gibco, Carlsbad, CA, USA), 10% of FBS (Gibco), 100 U/mL penicillin, and 100 μg/ mL streptomycin. Cells were incubated at 37° C in a humidified atmosphere of 5% CO2. For sublethal heat treatment, 4T1 cells were then incubated at 45° C for 10 minutes (HT=high temperature); cells incubated at 37° C for 10 minutes was regarded as control (NT=negative control).

### Colony formation assay

The sublethal heat treated 4T1 cells were seeded in a 6-well plate at a density of 500 cells/well then cultured at 37° C in a 5% CO_2_ humidified atmosphere. The medium was changed every the other day during 7 days of culture. Then the cells were washed twice with PBS. After that, cells were fixed in 4% paraformaldehyde for 20 min, stained with 1% crystal violet for 30 min at room temperature, washed again and photographed.

### Transwell invasion assay

Transwell chambers with 8 μm pores (Costar, Corning, NY, USA) were used to perform invasion assays. Matrigel (BD Biosciences, NJ, USA) was coated on the top side of the inserts. Transwell invasion assay was performed on 4T1 cells and HUVECs. HUVECs were pretreated with supernatants of sublethal heat treated 4T1 cells culture. The upper chamber was filled with 200 μl serum-free medium, and 1×10^4^ 4T1 cells were seeded, while 600 μl medium with 5% FBS was added to the lower chamber. The chambers were maintained at 37° C in 5% CO_2_ for 24h. Then, cells on the upper chamber were removed by cotton swabs. The inserts were then fixed in 4% paraformaldehyde for 20 min and stained with 1% crystal violet for 30 min. The invaded cells on the bottom of the membrane were assessed using a microscope and photographed. All experiments were performed in triplicate.

### Tumor graft

After sublethal heat treatment, 1×10^7^ 4T1 cells were re-suspended in 10 μL of DMEM medium and then drawn into a 20 μL Hamilton syringe with a 30-gauge needle and injected subcutaneously into the BALB/C nude mice (each group four mice, 4-6 weeks). The growth of the subcutaneous tumors was determined by measuring tumor length (L) and width (W) every week and the tumor volumes were calculated by the formula: V = (L × W^2^)/2. All mice were sacrificed at the end of the 3^rd^ week. All animal experiments in this study were performed in accordance with guidelines approved by Animal Care and Use Committee of Ruijin hospital.

### Immunohistochemistry (IHC)

Four protein expressions (Heat shock protein 1B, NOB1, BCLAF1 and CRIP1) were measured by immunohistochemistry. The subcutaneous tumors of the nude mice were fixed by 4% phosphate-buffered paraformaldehyde for 24 hours. The specimens were embedded in paraffin and sectioned into 5-μm thick sections. The tissue sections were deparaffinized and incubated in 0.05% trypsin at 37° C for 30 min, which was followed by peroxidase blocking to retrieve antigens and incubation with primary antibodies-Heat shock protein 1B (1:200, NOVUS, NBP2-16896), NOB1 (1:200, Abcam, ab224619), BCLAF1 (1:200, Abcam, ab181240), CRIP1 (1:200, Abcam, ab167087) at 4° C overnight. The sections were then incubated with HRP-secondary antibody (Fuzhou Maixin Biotech. Co., Ltd, MaxvisionTM2 HRP-Polymer anti-Mouse IHC Kit, KIT-5902) at 37° C for 2 hours and detected using a DAB Kit (Fuzhou Maixin Biotech. Co. Ltd, DAB Kit, DAB-0031). Then, the slides were mounted with neutral resin and coverslipped. After staining, the sections were observed under light microscopy (Zeiss, Axio Imager A2). These protein expressions were semi-quantitatively evaluated in representative tumor area. The staining intensity was defined as: negative-0, moderate-1, strong-2; the staining percentage was scored as: 0%-0, 1~25%-1,26~50%-2, 51~75%-3, 76~100%-4. The scores of intensity and percentage were multiplied to get a final score of 0 to 8. The total expressions of these proteins were determined as: negative, low expression (score<4), high expression (score≥4).

### Protein preparation

Cell lysis was performed with lysis buffer [8 M urea, 2 mM ethylene diamine tetra-acetic acid (EDTA), 10 mM dithiothreitol (DTT), and 1% protease inhibitor cocktail III]. The remained debris was removed by centrifugation (20,000 g, 4° C, and 10 min). Finally, the proteins were precipitated with cold 15% trichloroacetic acid (TCA; 2 h, and − 20° C). After centrifugation (4° C, 10 min), the supernatant was discarded. The remained precipitate was washed with cold acetone for three times. Proteins were redissolved in the buffer [8 M urea, 100 mM tetraethyl ammonium bromide (TEAB), pH 8.0], and the protein concentration was determined with BCA Protein Assay kit according to the manufacturer’s instructions.

### FASP enzymolysis of protein

Take appropriate amount of samples, add 1m DTT solution to the final concentration of 100mm, and incubate at 56° C for 1 hour. Take 200 μg of each sample, add 200 μl UA buffer (8m urea, 150mm Tris HCl, pH 8.5, remove low molecular weight impurities with UA, including SDS) and mix well, then transfer to 10kd ultrafiltration centrifuge tube, centrifugation 14000g for 15min. Add 200μl UA buffer, centrifuge 14000g for 15min, and discard the filtrate. Add 100μl IAA (50mm IAA in UA), oscillate at 600 rpm for 1 min, keep away from light for 30 min, and centrifugate at 14000 g for 10 min. Add 100ul UA buffer, centrifugate 14000g for 10min, and repeat twice. Add 100μl 50mm NH4HCO3 solution, centrifuge 14000g for 10min, and repeat twice. Add 40μl trypsin buffer (5μg trypsin in 40 μl 50mm NH_4_HCO_3_ solution), oscillate at 600 rpm for 1 min, and 37° C for 16-18 hours. Replace the collecting tube, centrifugate 14000 g for 10 min, add 25μl 25 mm NH4HCO3 solution, oscillate at 600 rpm for 1 min, centrifuge for 10 min, repeat once, combine filtrate, freeze-drying, add 50μl 0.1% TFA for dissolution, quantitative analysis of peptide segments by fluorescence method, desalting with rp-c18 solid phase extraction column (equilibrium: 1ml methanol (containing 0.1% TFA) washing once, 90% acetonitrile water 1ml (containing 0.1% TFA) washing once, water (containing 0.1% TFA) washing once for 1 time; adsorption sample: 1 ml of water (containing 0.1% TFA) is added to the sample to fully dissolve the sample, and the sample is naturally adsorbed by gravity for 3 times; washing: washing with 0.1% trifluoroacetic acid water for 3 times; elution: using 90% acetonitrile water (containing 0.1% TFA) for natural elution by gravity for 3 times. Re dissolution: vacuum drying, 0.1% formic acid water re dissolution sample, after mass spectrometry analysis, mass spectrometry analysis.

### LCMS / MS analysis of enzymolysis products

LCMS/MS analysis of enzymolysis products: according to the quantitative results, 1μg of enzymolysis products were taken for LC-MS/MS analysis, and each sample was analyzed once. The separation was carried out by a nanoliter flow rate HPLC system easy-nLC1200. Liquid a was 0.1% formic acid water solution, and solution B was 0.1% formic acid-80% acetonitrile solution. The sample was loaded with an automatic sampler and separated by an analysis column (75um * 15cm, in house packed with C18-AQ1.9 μm) at a flow rate of 300nl/min. The related liquid phase gradients were as follows: 0-18 minutes, liquid B linear gradient from 4% to 8%; 18 minutes to 85 minutes, liquid B linear gradient from 8% to 22%; 85 minutes to 110 minutes, liquid B linear gradient from 22% to 45%; 110 minutes to 114 minutes, liquid B linear gradient from 45% to 100%; 114 minutes to 120 minutes, liquid B maintained at 100%. The samples were cleaned with blank solvent for 54 min. The hydrolysates were separated by capillary high performance liquid chromatography (HPLC) and analyzed by QE-HF mass spectrometer (Thermo Fisher). Analysis time: 120min, detection method: positive ion, spray voltage: 1.9kV, ion transfer capillary temperature: 275 degrees C, corrected by standard correction solution before use, mother ion scanning range: 350-1600 m/z, the mass charge ratio of fragments of polypeptide and polypeptide is collected according to the following methods: data dependent scanning mode, fragmentation mode: collision induced dissociation (HCD, high energy) The normal energy was 30%, and the dynamic exclusion time was 30 s. The resolution of MS1 is 120000 at M/Z 400, the AGC value is set to 3e6, the resolution of MS2 is 15000 at M/Z 400, the AGC is set to 1E5, and the maximum ion accumulation time is 45 Ms. The profile mode was used for the first mass spectrometry, and centroid mode was used for the second mass spectrometry to reduce the data file size.

### Unmarked analysis of MaxQuant

LC-MS / MS original files were imported into MaxQuant software (version No. 1.6.0.1) for database search. The search engine was Andromeda, and LFQ non-standard quantitative analysis was conducted. The database was downloaded from UniProt (uniprot-mouse-85390-20190524.fasta, including 85390 sequences, downloaded from May 24, 2018), The reverse Library UniProt of mouse is used to calculate the false positive rate (FDR) of peptide and protein. MaxQuant software integrates LFQ algorithm by extracting the isotope peak of each peptide in each analysis. MaxQuant platform calculates protein ratio by using the median value of the ratio of common peptides in all analyses, which represents a fairly approximate estimation of protein ratio. From MaxQuant analysis “peptides.txt ” and “proteinGroups.txt” the file was imported into Perseus (version 1.5.1.6) software for further analysis, and the site, reverse database and common contaminant protein library were filtered out The data were grouped and some null values which did not meet the analysis standard were eliminated.

### Bioinformatics analysis of differentially expressed proteins (DEPs)

GO annotation of DEPs was derived from the UniProt-GOA database (http://www.ebi.ac.uk/GOA/). DEPs were classified by GO annotation based on three categories, including biological processes (BPs), cellular compartments (CCs), and molecular functions (MFs). KEGG pathway analysis of DEPs was performed with KOBAS online analysis database. Protein-protein interaction (PPI) network of DEPs was constructed with online STRING database (https://string-db.org), and an interaction with a combined score >0.4 was considered as statistical significance. Cytoscape, an open source bioinformatic software platform, was used to visualize molecular interaction networks. The plug-in Molecular Complex Detection (MCODE) in Cytoscape software was used to cluster a given network based on topology to find densely connected regions. The PPI networks were drawn with Cytoscape, and the most significant modules in the PPI networks were identified with MCODE method with the default criteria, including MCODE score>5, degree cutoff value=2, node score cutoff value=0.2, Maxdepth=100, and k-score=2.

### Statistical analysis

The data were analyzed using Statistical Program for Social Sciences 19.0 software (SPSS, Chicago, IL, USA) and GraphPad Prism 5.0 (GraphPad Software, LaJolla, CA, USA). Data were presented as mean±SD and comparisons were calculated by Student’s t-test (two-sided, unpaired). All experiments were repeated at least three times. P<0.05 was considered to indicate a statistically significant difference.

## Supplementary Material

Supplementary Figure 1

Supplementary Table 1

Supplementary Tables 2 and 3

Supplementary Table 4

Supplementary Table 5

Supplementary Tables 6 and 7

Supplementary Table 8

Supplementary Table 9
