# Supplementary material for: Sublethal heat treatment promotes breast cancer metastasis and its molecular mechanism revealed by quantitative proteomic analysis
Source: Aging (Albany NY). 2022 Feb 12;14(3):1389–406. doi: 10.18632/aging.203884 (PMC8876919; doi:10.18632/aging.203884)
Supplement: Supplementary Figure 1 [file aging-14-203884-s001.pdf]

SUPPLEMENTARY FIGURE

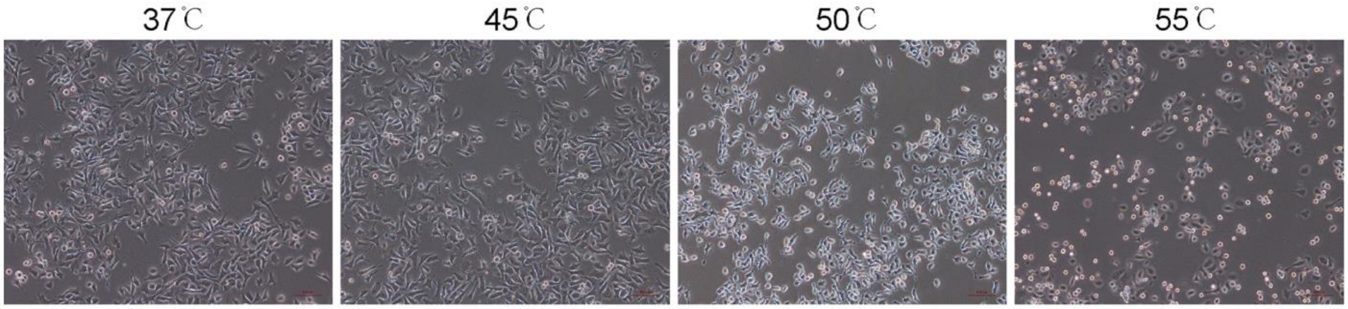

Supplementary Figure 1. The effects of different temperatures on the state of 4T1 cells were observed under microscope.
