# Supplementary material for: Sublethal heat treatment promotes breast cancer metastasis and its molecular mechanism revealed by quantitative proteomic analysis
Source: Aging (Albany NY). 2022 Feb 12;14(3):1389–406. doi: 10.18632/aging.203884 (PMC8876919; doi:10.18632/aging.203884)
Supplement: Supplementary Tables 2 and 3 [file aging-14-203884-s003.pdf]

Supplementary Table 2. GO.top.down.

Id	Term	Category	pval	Enrichment_score	Protein_gene
GO:0090410	malonate catabolic process	biological_process	0	23.1066666666667	Q3URE1:Acsf3
GO:0071577	zinc II ion transmembrane transport	biological_process	0	23.1066666666667	Q8C145:Slc39a6
GO:0006622	protein targeting to lysosome	biological_process	0	23.1066666666667	O35114:Scarb2
GO:1905123	regulation of glucosylceramidase activity	biological_process	0	23.1066666666667	O35114:Scarb2
GO:0010389	regulation of G2/M transition of mitotic cell cycle	biological_process	0	23.1066666666667	P51943:Ccna2; Q6PHZ2:Camk2d
GO:0044320	cellular response to leptin stimulus	biological_process	0	23.1066666666667	P51943:Ccna2
GO:0044843	cell cycle G1/S phase transition	biological_process	0	23.1066666666667	P51943:Ccna2
GO:0071314	cellular response to cocaine	biological_process	0	23.1066666666667	P51943:Ccna2
GO:0071373	cellular response to luteinizing hormone stimulus	biological_process	0	23.1066666666667	P51943:Ccna2
GO:1990314	cellular response to insulin-like growth factor stimulus	biological_process	0	23.1066666666667	P51943:Ccna2
GO:1990204	oxidoreductase complex	cellular_component	0	23.1066666666667	Q91VT4:Cbr4
GO:0097124	cyclin A2-CDK2 complex	cellular_component	0	23.1066666666667	P51943:Ccna2
GO:0000307	cyclin-dependent protein kinase holoenzyme complex	cellular_component	0	23.1066666666667	P61025:Cks1b
GO:0043194	axon initial segment	cellular_component	0	23.1066666666667	Q6PHZ2:Camk2d
GO:0042584	chromaffin granule membrane	cellular_component	0	23.1066666666667	Q6P069:Sri
GO:0020018	ciliary pocket membrane	cellular_component	0	23.1066666666667	Q9ERB0:Snap29
GO:0033179	proton-transporting V-type ATPase, V0 domain	cellular_component	0	23.1066666666667	P51863:Atp6v0d1
GO:0033181	plasma membrane proton-transporting V-type ATPase complex	cellular_component	0	23.1066666666667	P51863:Atp6v0d1
GO:0000220	vacuolar proton-transporting V-type ATPase, V0 domain	cellular_component	0	23.1066666666667	P15920:Atp6v0a2
GO:0097413	Lewy body	cellular_component	0	23.1066666666667	P11352:Gpx1
GO:0003955	NAD(P)H dehydrogenase (quinone) activity	molecular_function	0	23.1066666666667	Q91VT4:Cbr4
GO:0008753	NADPH dehydrogenase (quinone) activity	molecular_function	0	23.1066666666667	Q91VT4:Cbr4
GO:0047025	3-oxoacyl-[acyl-carrier-protein] reductase (NADH) activity	molecular_function	0	23.1066666666667	Q91VT4:Cbr4
GO:0070402	NADPH binding	molecular_function	0	23.1066666666667	Q91VT4:Cbr4
GO:0016878	acid-thiol ligase activity	molecular_function	0	23.1066666666667	Q3URE1:Acsf3
GO:0090409	malonyl-CoA synthetase activity	molecular_function	0	23.1066666666667	Q3URE1:Acsf3
GO:0016788	hydrolase activity, acting on ester bonds	molecular_function	0	23.1066666666667	Q9D7N9:Apmap
GO:0016844	strictosidine synthase activity	molecular_function	0	23.1066666666667	Q9D7N9:Apmap
GO:0097472	cyclin-dependent protein kinase activity	molecular_function	0	23.1066666666667	P51943:Ccna2
GO:0061575	cyclin-dependent protein serine/threonine kinase activator activity	molecular_function	0	23.1066666666667	P61025:Cks1b

Supplementary Table 3. GO.top.up.

Id	Term	Category	pval	Enrichment_score	Protein_gene
GO:0000301	retrograde transport, vesicle recycling within Golgi	biological_process	0	20.8795180722892	Q9QYE6:Golga5
GO:0007091	metaphase/anaphase transition of mitotic cell cycle	biological_process	0	20.8795180722892	A2A6Q5:Cdc27
GO:0070979	protein K11-linked ubiquitination	biological_process	0	20.8795180722892	A2A6Q5:Cdc27
GO:0032410	negative regulation of transporter activity	biological_process	0	20.8795180722892	Q99LJ0:Cttnbp2nl
GO:0034763	negative regulation of transmembrane transport	biological_process	0	20.8795180722892	Q99LJ0:Cttnbp2nl
GO:1902525	regulation of protein monoubiquitination	biological_process	0	20.8795180722892	Q8BH57:Wdr48
GO:0050806	positive regulation of synaptic transmission	biological_process	0	20.8795180722892	Q8BNY6:Ncs1
GO:1903262	negative regulation of serine phosphorylation of STAT3 protein	biological_process	0	20.8795180722892	Q99LL5:Pwp1
GO:2000738	positive regulation of stem cell differentiation	biological_process	0	20.8795180722892	Q99LL5:Pwp1
GO:0001763	morphogenesis of a branching structure	biological_process	0	20.8795180722892	E9Q5F9:Setd2
GO:0033185	dolichol-phosphate-mannose synthase complex	cellular_component	0	20.8795180722892	Q9D1Q4:Dpm3; O70152:Dpm1
GO:0030906	retromer, cargo-selective complex	cellular_component	0	20.8795180722892	P40336:Vps26a
GO:0000243	commitment complex	cellular_component	0	20.8795180722892	P62315:Snrpd1
GO:0005666	DNA-directed RNA polymerase III complex	cellular_component	0	20.8795180722892	P52432:Polr1c; Q923G2:Polr2h
GO:0099053	activating signal cointegrator 1 complex	cellular_component	0	20.8795180722892	E9PZJ8:Ascc3
GO:0005960	glycine cleavage complex	cellular_component	0	20.8795180722892	Q91WK5:Gcsh
GO:0005736	DNA-directed RNA polymerase I complex	cellular_component	0.000106104412585285	13.9196787148594	P52432:Polr1c; Q923G2:Polr2h
GO:0000788	nuclear nucleosome	cellular_component	0.000106104412585285	13.9196787148594	P84244:H3f3a; P84244:H3f3b
GO:0005876	spindle microtubule	cellular_component	0.000296003570783366	7.82981927710843	A2A6Q5:Cdc27; Q8BHJ5:Tbl1xr1; Q6P9P6:Kif11
GO:0001740	Barr body	cellular_component	0.000409697963046072	10.4397590361446	P84244:H3f3a; P84244:H3f3b
GO:1990889	H4K20me3 modified histone binding	molecular_function	0	20.8795180722892	Q99LL5:Pwp1
GO:0016831	carboxy-lyase activity	molecular_function	0	20.8795180722892	Q99K01:Pdxdc1
GO:0070699	type II activin receptor binding	molecular_function	0	20.8795180722892	Q9D6K5:Synj2bp
GO:0016706	oxidoreductase activity, acting on paired donors, with incorporation or reduction of molecular oxygen, 2-oxoglutarate as one donor, and incorporation of one atom each of oxygen into both donors	molecular_function	0	20.8795180722892	Q9JJF3:Riox1; Q80Y84:Kdm5b
GO:0051864	histone demethylase activity (H3-K36 specific)	molecular_function	0	20.8795180722892	Q9JJF3:Riox1
GO:0004827	proline-tRNA ligase activity	molecular_function	0	20.8795180722892	Q8CFI5:Pars2
GO:1990050	phosphatidic acid transporter activity	molecular_function	0	20.8795180722892	Q9D8Z2:Triap1
GO:0010521	telomerase inhibitor activity	molecular_function	0	20.8795180722892	Q9CZX5:Pinx1
GO:0033130	acetylcholine receptor binding	molecular_function	0	20.8795180722892	Q9CQU3:Rer1
GO:0047545	2-hydroxyglutarate dehydrogenase activity	molecular_function	0	20.8795180722892	Q91YP0:L2hgdh
