# Supplementary material for: Sublethal heat treatment promotes breast cancer metastasis and its molecular mechanism revealed by quantitative proteomic analysis
Source: Aging (Albany NY). 2022 Feb 12;14(3):1389–406. doi: 10.18632/aging.203884 (PMC8876919; doi:10.18632/aging.203884)
Supplement: Supplementary Tables 6 and 7 [file aging-14-203884-s006.pdf]

Supplementary Table 6. KEGG.top.up.

Id	Term	pval	Enrichment_score	Protein_gene
mmu00040	Pentose and glucuronate interconversions	0	22.9459459459459	P45377:Akr1b8
mmu03020	RNA polymerase	0.0000977738049485203	9.83397683397683	P60898:Polr2i; P52432:Polr1c; Q923G2:Polr2h
mmu05322	Systemic lupus erythematosus	0.00018944513170987	8.60472972972973	P62315:Snrpd1; P84244:H3f3a; P84244:H3f3b
mmu04623	Cytosolic DNA-sensing pathway	0.000296587969002751	11.472972972973	P52432:Polr1c; Q923G2:Polr2h
mmu04550	Signaling pathways regulating pluripotency of stem cells	0.000719146017537135	9.17837837837838	P23798:Pcgf2; Q63844:Mapk3
mmu00790	Folate biosynthesis	0.0018501233415561	11.472972972973	P45377:Akr1b8
mmu05034	Alcoholism	0.00296009673583045	4.58918918918919	P84244:H3f3a; P84244:H3f3b; Q63844:Mapk3
mmu04914	Progesterone-mediated oocyte maturation	0.00367511252103553	5.73648648648649	A2A6Q5:Cdc27; Q63844:Mapk3
mmu04666	Fc gamma R-mediated phagocytosis	0.00534748682732911	5.0990990990991	Q9D898:Arpc5l; Q63844:Mapk3
mmu03460	Fanconi anemia pathway	0.0053974672691678	7.64864864864865	Q8BH57:Wdr48
mmu00920	Sulfur metabolism	0.0053974672691678	7.64864864864865	Q80V26:Impad1
mmu01521	EGFR tyrosine kinase inhibitor resistance	0.0053974672691678	7.64864864864865	Q63844:Mapk3
mmu04620	Toll-like receptor signaling pathway	0.0053974672691678	7.64864864864865	Q63844:Mapk3
mmu04658	Th1 and Th2 cell differentiation	0.0053974672691678	7.64864864864865	Q63844:Mapk3
mmu04659	Th17 cell differentiation	0.0053974672691678	7.64864864864865	Q63844:Mapk3
mmu04664	Fc epsilon RI signaling pathway	0.0053974672691678	7.64864864864865	Q63844:Mapk3
mmu05216	Thyroid cancer	0.0053974672691678	7.64864864864865	Q63844:Mapk3
mmu05218	Melanoma	0.0053974672691678	7.64864864864865	Q63844:Mapk3
mmu05219	Bladder cancer	0.0053974672691678	7.64864864864865	Q63844:Mapk3
mmu05223	Non-small cell lung cancer	0.0053974672691678	7.64864864864865	Q63844:Mapk3

Supplementary Table 7. KEGG.top.down.

Id	Term	pval	Enrichment_score	Protein_gene
mmu05032	Morphine addiction	0.00000119374883178	13.8048780487805	Q3UYH7:Adrbk2; P68181:Prkacb; Q9CXP8:Gng10; P63213:Gng2
mmu04340	Hedgehog signaling pathway	4.71123465396585E-06	15.530487804878	Q3UYH7:Adrbk2; P68181:Prkacb; Q8BVP5:Csnk1g2
mmu04740	Olfactory transduction	4.71123465396585E-06	15.530487804878	Q3UYH7:Adrbk2; Q6PHZ2:Camk2d; P68181:Prkacb
mmu04062	Chemokine signaling pathway	6.54076473409003E-06	8.62804878048781	Q3UYH7:Adrbk2; P68181:Prkacb; Q9CXP8:Gng10; P63213:Gng2; P60766:Cdc42
mmu05034	Alcoholism	0.0000317737498516404	6.90243902439024	Q9CXP8:Gng10; P62141:Ppp1cb; P63213:Gng2; P27661:H2afx; Q9QZQ8:H2afy
mmu04142	Lysosome	0.0000433809350810006	8.28292682926829	O35114:Scarb2; P51863:Atp6v0d1; P15920:Atp6v0a2; P22892:Ap1g1
mmu04725	Cholinergic synapse	0.0000433809350810006	8.28292682926829	Q6PHZ2:Camk2d; P68181:Prkacb; Q9CXP8:Gng10; P63213:Gng2
mmu04728	Dopaminergic synapse	0.0000490250173998093	6.47103658536585	Q6PHZ2:Camk2d; P68181:Prkacb; Q9CXP8:Gng10; P62141:Ppp1cb; P63213:Gng2
mmu04723	Retrograde endocannabinoid signaling	0.0000511543321342115	4.67584579071597	Q9DCS9:Ndufb10; Q9ERS2:Ndufa13; P68181:Prkacb; Q9CXP8:Gng10; Q9Z1P6:Ndufa7; P63213:Gng2; Q9CQ91:Ndufa3
mmu05031	Amphetamine addiction	0.0000658055331679858	10.3536585365854	Q6PHZ2:Camk2d; P68181:Prkacb; P62141:Ppp1cb
mmu04713	Circadian entrainment	0.0000767057005718373	7.52993348115299	Q6PHZ2:Camk2d; P68181:Prkacb; Q9CXP8:Gng10; P63213:Gng2
mmu05200	Pathways in cancer	0.000132144111739917	4.14146341463415	P61025:Cks1b; Q6PHZ2:Camk2d; P68181:Prkacb; Q61301:Ctnna2; Q9CXP8:Gng10; P63213:Gng2; P60766:Cdc42
mmu05323	Rheumatoid arthritis	0.000148173422729048	8.87456445993031	P51863:Atp6v0d1; P15920:Atp6v0a2; P28862:Mmp3
mmu04724	Glutamatergic synapse	0.000198766821672066	6.37148217636023	Q3UYH7:Adrbk2; P68181:Prkacb; Q9CXP8:Gng10; P63213:Gng2
mmu05203	Viral carcinogenesis	0.000277018125999705	4.93031358885017	P51943:Ccna2; P51863:Atp6v0d1; A2AN08:Ubr4; P68181:Prkacb; P60766:Cdc42
mmu04750	Inflammatory mediator regulation of TRP channels	0.000285983119188843	7.76524390243902	Q6PHZ2:Camk2d; P68181:Prkacb; P62141:Ppp1cb
mmu04727	GABAergic synapse	0.000496777879623897	6.90243902439024	P68181:Prkacb; Q9CXP8:Gng10; P63213:Gng2
mmu05165	Human papillomavirus infection	0.000497925231416475	3.88262195121951	P51943:Ccna2; P51863:Atp6v0d1; P15920:Atp6v0a2; A2AN08:Ubr4; P68181:Prkacb; P60766:Cdc42
mmu04912	GnRH signaling pathway	0.000799042509319161	6.21219512195122	Q6PHZ2:Camk2d; P68181:Prkacb; P60766:Cdc42
mmu04926	Relaxin signaling pathway	0.000799042509319161	6.21219512195122	P68181:Prkacb; Q9CXP8:Gng10; P63213:Gng2
